# Effects of current alcohol use on brain volume among older adults in the Gothenburg H70 Birth Cohort study 2014–16

**DOI:** 10.1007/s00406-023-01691-x

**Published:** 2023-09-19

**Authors:** Olof Lindberg, Felicia Ahlner, Theofanis Tsevis, Joana B. Pereira, Eric Westman, Ingmar Skoog, Lars-Olof Wahlund

**Affiliations:** 1 Division of Clinical Geriatrics, Department of Neurobiology, Care Sciences, and Society, Karolinska Institutet, Neo Floor 7 SE, 141 83, Huddinge, Stockholm, Sweden; 2 Neuropsychiatric Epidemiology, Institute of Neuroscience and Physiology, Department of Psychiatry and Neurochemistry, Centre for Ageing and Health (AgeCap), Sahlgrenska Academy at the University of Gothenburg, Gothenburg, Sweden; 3 Department of Neuroimaging, Centre for Neuroimaging Sciences, Institute of Psychiatry, Psychology and Neuroscience, King’s College London, London, UK; 4 Region Västra Götaland, Sahlgrenska University Hospital, Psychiatry, Cognition and Old Age Psychiatry Clinic, Gothenburg, Sweden

**Keywords:** Alcohol consumption, Elderly, Population-based, Brain changes, Diffusion tensor, Structural magnetic resonance images

## Abstract

Brain gray- and white matter changes is well described in alcohol-dependent elderly subjects; however, the effect of lower levels of alcohol consumption on the brain is poorly understood. We investigated the impact of different amounts of weekly alcohol consumption on brain structure in a population-based sample of 70-year-olds living in Gothenburg, Sweden. Cross-sectional data from 676 participants from The Gothenburg H70 Birth Cohort Study 2014–16 were included. Current alcohol consumers were divided into seven groups based on self-reported weekly amounts of alcohol consumption in grams (g) (0–50 g/week, used as reference group, 51–100 g/week, 101–150 g/week, 151–200 g/week, 201–250 g/week, 251–300 g/week, and > 300 g/week). Subcortical volumes and cortical thickness were assessed on T1-weighted structural magnetic resonance images using FreeSurfer 5.3, and white matter integrity assessed on diffusion tensor images, using tract-based statistics in FSL. General linear models were carried out to estimate associations between alcohol consumption and gray- and white matter changes in the brain. Self-reported consumption above 250 g/week was associated with thinning in the bilateral superior frontal gyrus, the right precentral gyrus, and the right lateral occipital cortex, in addition to reduced fractional anisotropy (FA) and increased mean diffusivity (MD) diffusively spread in many tracts all over the brain. No changes were found in subcortical gray matter structures. These results suggest that there is a non-linear relationship between alcohol consumption and structural brain changes, in which loss of cortical thickness only occur in non-demented 70-year-olds who consume more than 250 g/week.

**Supplementary Information:**

The online version contains supplementary material available at 10.1007/s00406-023-01691-x.

## Introduction

Heavy alcohol consumption is associated with accelerated brain damage [8, 27]. Evidence from multiple structural magnetic resonance imaging (MRI) studies show that reduced gray and white matter volume can be observed in the brains of patients with alcohol-related syndromes [42, 43]. In patients with alcohol use disorders (AUD) without cognitive deficits, findings are more inconsistent. Most studies have found atrophy in the prefrontal cortex (PFC) [5, 13, 23, 28, 37], but other brain regions have been implicated [18, 24, 25, 43]. Moreover, diffusion tensor imaging (DTI) studies have shown reduced white matter integrity in several brain regions among patients with AUD [43]. Some studies found that damage may be more severe in anterior compared to posterior white matter [43].

In contrast to chronic heavy consumption, lower levels of alcohol use have been suggested to be harmless or even beneficial in relation to brain atrophy in older adults. However, several studies have questioned these findings [30, 38] and recent evidence on all-cause mortality has prompted non-use to be the safest level of consumption for all ages [15]. Thus, the current literature on this subject is inconsistent. Few studies have investigated the effect of moderate drinking on brain structure in general, and among older adults in particular [39]. Previous findings include associations between moderate alcohol consumption and reduced total brain and hippocampal volume [38], increased ventricle size [9], reduced gray matter density, and white matter damage [4, 14]. Other studies have found no associations [16], inverse associations [7], or associations only at higher consumption levels [22].

The inconsistencies in previous studies may partly be explained by differences in sample distributions of sex, age, and neurological diseases. Several studies suggest that women are at greater risk for negative consequences of alcohol use [1, 2, 19, 28].

However, reverse findings with more volume loss in men compared with women with AUD have also been reported [31].

Another confounding factor is differences in age between different samples. Aging is associated with structural changes to the brain [32], which may lead to differences among studies with different sample age. Furthermore, beyond cognitive disability induced by alcohol, cognitive status may also reflect a neurodegenerative condition that potentially cause another pattern of brain atrophy and be a confounding effect when investigating the alcohol–brain relationship.

Alcohol use is increasing in recent cohorts of older adults in many countries [3, 6, 17, 26]. Considering the demographic shift with aging populations worldwide, more research is needed to better understand the effects of alcohol on the brain in older adults. While the association between alcohol dependence and brain changes is well described [21], the effect of non-dependent alcohol consumption on the brain is poorly understood. More specifically, few studies have examined the association between alcohol consumption and brain structure in general populations of older adults representing normal aging with true distributions of sex, cognitive status, genetic variants, and alcohol consumption patterns.

The aim of this study is to investigate the association between current alcohol use and brain structure in a large population-based sample of 70-year-olds from the Gothenburg H70 Birth Cohort study.

## Methods

### Participants

Cross-sectional data were derived from the baseline examination of Birth cohort 1944 in the Gothenburg H70 Birth Cohort study 2014–16 (*n* = 1203, response rate 72.2%). Participants were systematically selected based on birth dates obtained from the Swedish Tax Agency. The study comprises a one-day general health examination and several additional examinations (e.g., brain imaging). Study procedures have been described in detail elsewhere [33].

A total of 676 participants had a good-quality magnetic resonance imaging (MRI) [34] and available data on alcohol consumption. Out of these, 555 participants were cognitively unimpaired according to the Clinical Dementia Rating (CDR = 0) scale [20], while 121 had CDR > 0. A separate analysis was performed for the cognitively unimpaired subjects.

Ethical permits were obtained from the Swedish Ethical Review Authority (Number 869-13 and by the Radiation Protection Committee Approval number: 13-64). Written informed consents were obtained prior to participation in the study. All methods were carried out in accordance with relevant guidelines and regulations.

### Assessment of alcohol consumption

Information on alcohol consumption was assessed in a face-to-face interview during the general health examination. Alcohol variables included weekly consumption of beer, wine, and spirits during the past month. Separate questions for each beverage were used to reduce the risk of underreporting [11]. Total weekly consumption (grams per week [g/week]) was calculated based on amounts reported and categorized into seven subgroups: 0–50 g/week, 51–100 g/week, 101–150 g/week, 151–200 g/week, 201–250 g/week, 251–300 g/week, and above 300 g/week. The 0–50 g/week group was considered as a reference group.

### MRI acquisition

The participants were scanned on a 3.0 T Philips Achieva system (Philips Medical Systems), using a 3D T1-weigthed Turbo Field Echo (TFE) sequence (Repetition time (RT) = 7.2 ms, Echo time (TE) = 3.2 ms, flip angle = 9°, matrix size = 250 × 250 mm, field of view = 256 × 256, slice thickness = 1.0 mm) and diffusion-weighted sequence (encoded with 1 *b* value shell: 800ks/mm^2^, along with 32 directions and 1 *b* = 0 image (RT = 7340 ms, ET = 83 ms, flip angle = 90°, matrix size = 112 × 112 mm, field of view = 224 × 224, slice thickness = 3.0 mm).

### MRI analysis

Cortical reconstruction and volumetric segmentation of subcortical volumes were performed on the T1 3D images using FreeSurfer’s 5.3 image analysis pipeline, which is documented and freely available for download online (http://surfer.nmr.mgh.harvard.edu/). The technical details of these procedures are described in prior publications, which are listed at https://surfer.nmr.mgh.harvard.edu/fswiki/FreeSurferMethodsCitation. Briefly, the whole-brain T1-weighted images underwent a correction for intensity homogeneity, skull striping, and segmentation into GM and white matter (WM). Cortical thickness was measured as the distance from the gray/white matter boundary to the corresponding pial surface. Subcortical segmentation and assessment of intracranial volume was also performed in FreeSurfer. Reconstructed data sets were visually inspected for accuracy, and segmentation errors were corrected. Quality control was carried out on all MRI data according to previous described procedures [34], and data management and processing were done through our database system [29].

Diffusion-weighted images were analyzed using the FMRIB’s Diffusion Toolbox from FSL (https://fsl.fmrib.ox.ac.uk/fsl/fslwiki) (Behrens et al., 2007). First, the data were corrected for distortions caused by eddy currents and head motion using the b0 non-diffusion data as a reference volume (Andersson and Skare, 2002). The resulting images were skull-striped and a diffusion tensor model was fitted at each voxel to determine the preferred diffusion direction as the principal eigenvector of the eigenvalue decomposition (Pierpaoli and Basser, 1996; Song et al., 2002). To provide information on the microstructural organization of the white matter, for each voxel the fractional anisotropy (FA) and mean diffusivity (MD) maps were computed (Beaulieu and Allen, 1994). The FA maps were transformed into MNI space using the tract-based spatial statistics tool. After normalization, FA images were resampled and subsequently merged into a single file to create a mean FA image for all subjects, which was then used to create a mean FA ‘skeleton’. The threshold of the skeleton was set to 0.2 to include the WM tracts that were common to all subjects. Individual FA maps were then projected onto this mean FA skeleton. The transformation matrix of FA obtained in the above steps was applied to MD maps.

### Statistical methods

#### Structural MRI

Analyses were performed on the total sample (*n* = 676). Group cortical thickness comparisons were performed using vertex-based GLM (general linear model) in FreeSurfer that included degree of weekly consumption in 50 g intervals and sex as factors and cortical thickness as dependent variable. The Gaussian smoothing kernel was 10 mm. The level of statistical significance was evaluated using a cluster-wise P (CWP) value correction procedure for multiple comparisons based on a Monte Carlo z-field simulation with a cluster forming threshold of *p* < 0.05 (vertex-z-threshold = 1.3). The association between FreeSurfer segmented subcortical volumes and alcohol consumption was assessed using univariate general linear models (GLM) in Statistica (TIBCO Software Inc. version 13. http://tibco.co), correcting for sex and intracranial volume.

#### Diffusion tensor imaging

To assess the relationship between alcohol consumption with FA and MD maps, voxel-wise regression analyses were carried out including the alcohol consumption as dependent variable and sex as a confounder. Moreover, group comparisons were carried out assessing the difference in FA and MD between the reference group and other groups with increasing alcohol consumption. All analyses were performed using the randomize tool of FSL with 5000 permutations. The results were corrected for multiple comparisons using threshold-free cluster enhancement corrections (*p* < 0.05).

## Results

### Sample characteristics

Characteristics of the sample of current drinkers (*n* = 676) are given in Table 1. The mean (SD) age of the sample at time of MRI scan was 70.5 (0.28), and 47.8% (*n* = 324) were men. A total of 302 participants (44.6%) were in the 0–50 g/week consumption group, 155 (22.9%) in the 51–100 g/week, 94 (13.9%) in the 101–150 g/week, 56 (8.3%) in the 151–200 g/week, 25 (3.7%) in the 201–250 g/week, 16 (2.4%) in the 251–300 g/week, and 28 (4.1%) in the > 300 g/week group. Only three women consumed more than 300 g pure alcohol per week, which were considered too few to be included in the analysis. Thus, men in the reference group were compared with men in the > 300 g/week-group (*n* = 25).

**Table 1:** The demographic characteristics according to categories of total alcohol intake

n T1 (n DTI)	0–50 g	51–100 g	101–150 g	151–200 g	201–250 g	251–300 g	≥ 300 g	Total
	*n* = 302 (290)	*n* = 155 (150)	*n* = 94 (89)	*n* = 56 (54)	*n* = 25 (22)	*n* = 16 (15)	*n* = 28 (26)	*n* = 676 (646)
% (no. of cases/total sample)								
Men	35.7 (108/302)	50.6 (78/155)*	59.6 (56/94)*	62.5 (35/56)*	48.0 (12/25)	62.5 (10/16)*	89.3 (25/28)*	47.9 (324/676)
Having partner	66.2 (200/302)	85.2 (132/155)*	84.0 (79/94)*	91.1 (51/56)*	72.0 (18/25)	81.3 (13/16)	82.1 (23/28)	76.3 (516/676)
Born in Sweden	78.8 (238/302)	90.3 (140/155)*	94.7 (89/94)*	89.3 (50/56)	92.0 (23/25)	100.0 (16/16)*	75.0 (21/28)	85.4 (577/676)^¶^
Smoking								
Current	6.6 (20/302)	7.7 (12/155)	9.6 (9/94)	7.1 (4/56)^b^	12.0 (3/25)^b^	12.5 (2/16)^b^	7.4 (2/27)^b^	7.7 (52/675)
Past	48.0 (145/302)	55.5 (86/155)	56.4 (53/94)	62.5 (35/56)	64.0 (16/25)	43.8 (7/16)	71.4 (20/27)*	53.6 (362/675)
Non-smoker	45.4 (137/302)	36.8 (57/155)	34.0 (32/94)	30.4 (17/56)*	24.0 (6/25)	43.8 (7/16)	18.5 (5/27)^b^*	38.7 (261/675)
Have used illicit drugs	3.3 (10/299)	2.0 (3/152)^b^	5.3 (5/94)^b^	1.8 (1/55)^b^	0 (0/25)^b^	6.3 (1/16)^b^	7.1 (2/28)^b^	3.3 (22/669)
Education								
≤ Primary	13.2 (40/302)	10.3 (16/155)	10.6 (10/94)	3.6 (2/56)*	0 (0/25)^b^	0 (0/16)^b^	21.4 (6/28)	10.9 (74/676)^¶^
Secondary	12.6 (38/302)	18.8 (29/155)	9.6 (9/94)	7.1 (4/56)^b^	12.0 (3/25)^b^	25.0 (4/16)^b^	7.1 (2/28)^b^	13.2 (89/676)^¶^
Post secondary	43.4 (131/302)	31.6 (49/155)*	38.3 (36/94)	41.1 (23/56)	48.0 (12/25)	25.0 (4/16)^b^	28.6 (8/28)	38.9 (263/676)^¶^
Higher	30.8 (93/302)	39.4 (61/155)	41.5 (39/94)	48.2 (27/56)*	40.0 (10/25)	50.0 (8/16)	42.9 (12/28)	37.0 (250/676)^¶^
Religious	32.8 (96/293)	23.0 (35/152)*	17.6 (16/91)*	10.9 (6/55)*	20.8 (5/24)^b^	0 (0/16)^b^*	14.8 (4/27)^b^	24.6 (162/658)^¶^
APOE Ɛ4 carriers								
Heterozygous	34.2 (101/295)	26.8 (40/149)	26.9 (25/93)	27.8 (15/54)	20.0 (5/25)^b^	43.8 (7/16)	37.0 (10/27)	30.8 (203/659)
Homozygous	1.7 (5/295)	2.7 (4/149)^b^	4.3 (4/93)^b^	5.6 (3/54)	0 (0/25)^b^	12.5 (2/16)^b^	0 (0/27)^b^	2.7 (18/659)
CDR ≥ 0.5	19.5 (59/302)	15.6 (24/154)	13.8 (13/94)	16.1 (9/56)	16.0 (4/25)^b^	12.5 (2/16)^b^*	32.1 (9/28)	17.8 (120/675)^¶^

^a^Chi-square tests and Kruskal–Wallis tests for categorical and continuous variables, respectively

^b^Fishers exact test was used to test sex differences

*Statistical significance *p* < .05

^¶^Sex differences (*p* < 0.05)

APOE Ɛ4 carrier status was not associated with more cortical thinning in subgroups that consumed more alcohol than the reference group. Smoking was not associated with decrease or increase of cortical or subcortical volume and was therefore not included in the model.

The present study comprised 352 women and 324 men. We did, however, not observe any significant interaction (gender*consumption; supplementary Fig. 1) in the analysis we performed. We, therefore, did not perform stratified analysis with men and women separately.

Within the reference group, individuals reporting no alcohol consumption during the past month (*n* = 80), were less likely to be in a relationship (*p* < 0.001), were less likely born in Sweden (*p* < 0.001), and had higher frequency of mild cognitive problems (CDR = 0.5; *p* = 0.006). There was, however, no significant difference between participants consuming 0 g compared with participants who consumed 1–50 g in brain volume or DTI measures investigated in this study. Therefore 0 g and 1–50 g were merged into one group.

### Associations between alcohol consumption and cortical thickness

**Fig. 1:**
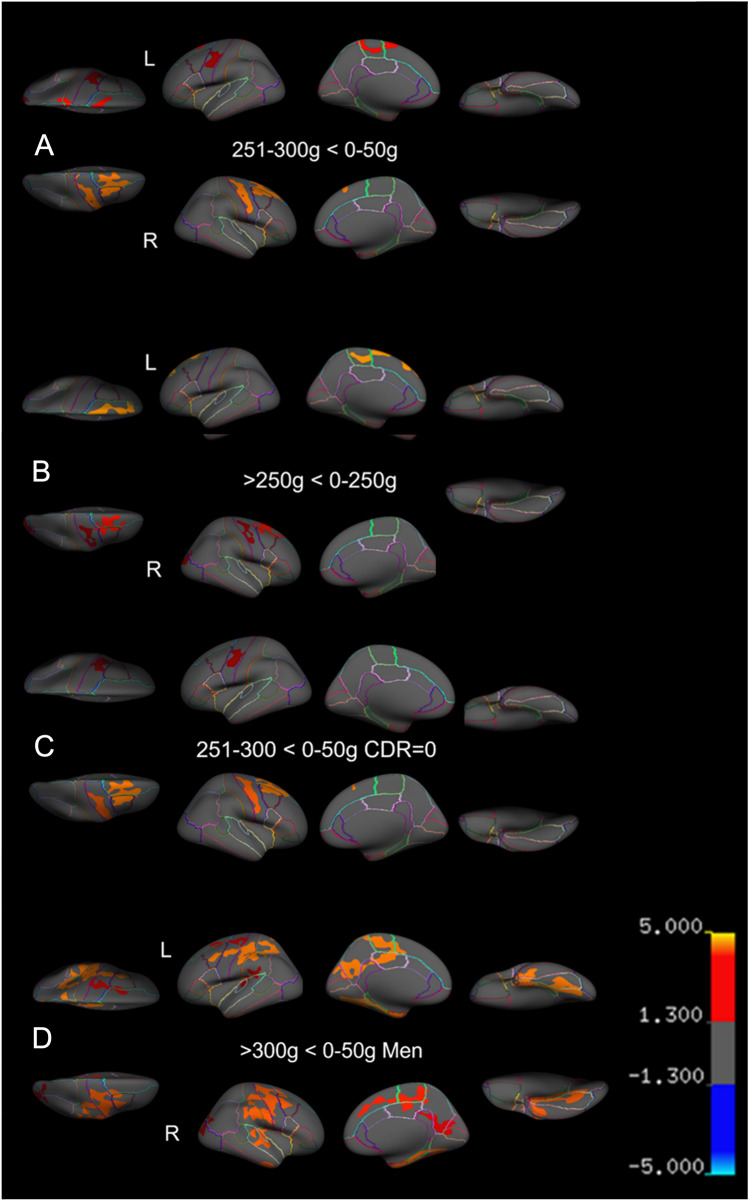
Cortical thinning in high-consuming participants. **A** Participants that consumed 0–50 g have thicker cortex than participants who consumed 251–300 g. **B** Participants who consumed less than 251 g have thicker cortex than participants who consumed more than 250 g. **C** Participants with CDR = 0, participants who consumed 0–50 g have thicker cortex than participants who consumed 251–300 g. **D** Men consumed 0–50 g/week have ticker cortex than men that consumed more than 300 g/week. Cortical thinning displayed on the inflated cortical surface. Warmer colors indicate that the reference group have thicker cortex. Images in the upper row display the left hemisphere, the lower column the right hemisphere. Cluster-wise *p* values < 0.05 are displayed. *L* left, *R* right

There were no differences regarding regional cortical thickness between the reference group and consumption groups < 250 g/week (i.e., 51–100 g/week, 101–150 g/week, 151–200 g/week, 201–250 g/week). In participants consuming 251–300 g/week (*n* = 16), atrophy was observed in the left precentral gyrus left superior frontal and left lateral occipital gyrus, the right postcentral gyrus, right caudal middle frontal gyrus and the right superior frontal gyrus, compared with the reference group (Fig. 1A; Supplementary Table 1).

**Fig. 2:**
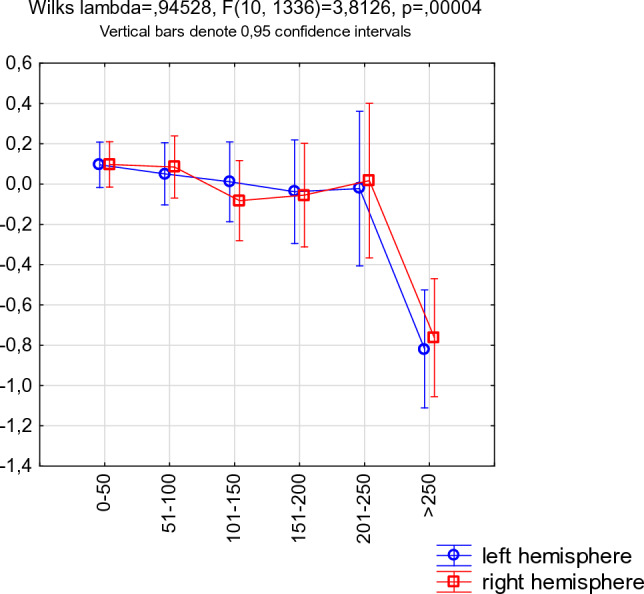
Cortical thinning in participants who are consuming > 250 g/week. The graph displays mean cortical thinning under areas that were reduced in participants who consumed > 250 g/week (areas with significant cortical thinning in Fig. 1B). The x-axis denotes alcohol consumption. The y-axis cortical thickness converted to z-scores

The results above indicate that cortical thinning may occur in participants who drink > 250 g/week. We, therefore, also compared all participants consuming ≤ 250 g/week (*n* = 632) with participants consuming > 250 g (*n* = 44). Participants consuming > 250 g/week displayed cortical thinning in the bilateral superior frontal gyrus, the right caudal middle frontal, precentral and lateral occipital gyrus (Fig. 1B, Fig. 2; supplementary table 2).

Cognitively unimpaired participants who consumed 251–300 g/week displayed cortical atrophy in the left precentral gyrus as well as the right precentral and caudal middle frontal and superior frontal gyrus compared to the reference group (Fig. 1C, supplementary table 3). No changes were observed in white matter or subcortical volumes.

In men consuming > 300 g/week (*n* = 25), cortical thinning was observed in many cortical areas (i.e., areas in the bilateral caudal superior and middle frontal gyrus, pre- and postcentral gyrus, entorhinal and fusiform gyrus, posterior cingulate gyrus, paracentral lobule) compared to the men in the reference group (*n* = 25; Fig. 1D, Supplementary Table 4).

### The association between alcohol consumption and subcortical volumes

Subcortical structures did not display reduced volume at any levels of alcohol consumption compared to the reference group.

### The association between alcohol consumption and white matter integrity

**Fig. 3:**
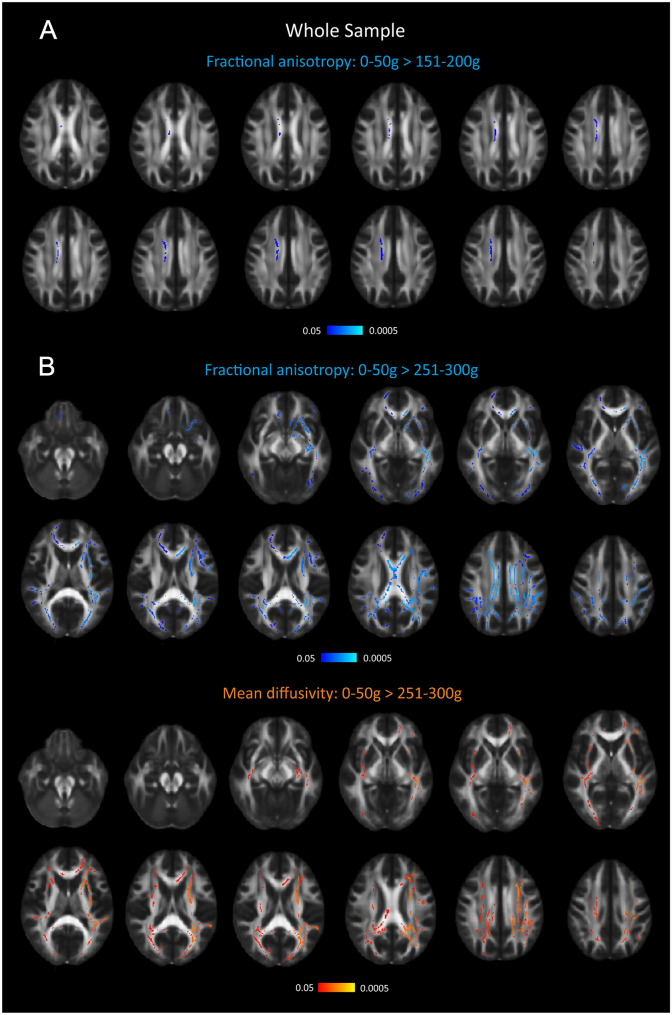
The reduction of fractional anisotropy and increase of mean diffusivity in participants who consumed 151–200 g/week and 251–300 g/week. **A** Reduced FA and in participants who consumed 151–200 g/week compared with participants who consumed 0–50 g/week, **B** Reduced FA and increased MD in participants who consumed 251–300 g/week compared with participants who consumed 0–50 g/week, Cold colors, areas of significant reduction, darkest blue = *p* < 0.05 brightest blue = *p* < 0.005. Warm colors, areas of significant increase, darkest red = *p* < 0.05 brightest red = *p* < 0.005

There were no differences in either FA or MD between reference group and consumption groups ≤ 151 g/week (51–100 g/week and 101–150 g/week). In participants consuming 151–200 g/week (*n* = 50), reduced FA was found in the body of the corpus callosum and in the superior corona radiata (Fig. 3A, Supplementary Table 5). In participants consuming 251–300 g/week (*n* = 15), FA was reduced, and MD was increased in a large number of white matter tracts (Fig. 3B, Supplementary Table 5). Men consuming > 300 g/week did not display reduced FA or increased MD in any white matter tract.

**Fig. 4:**
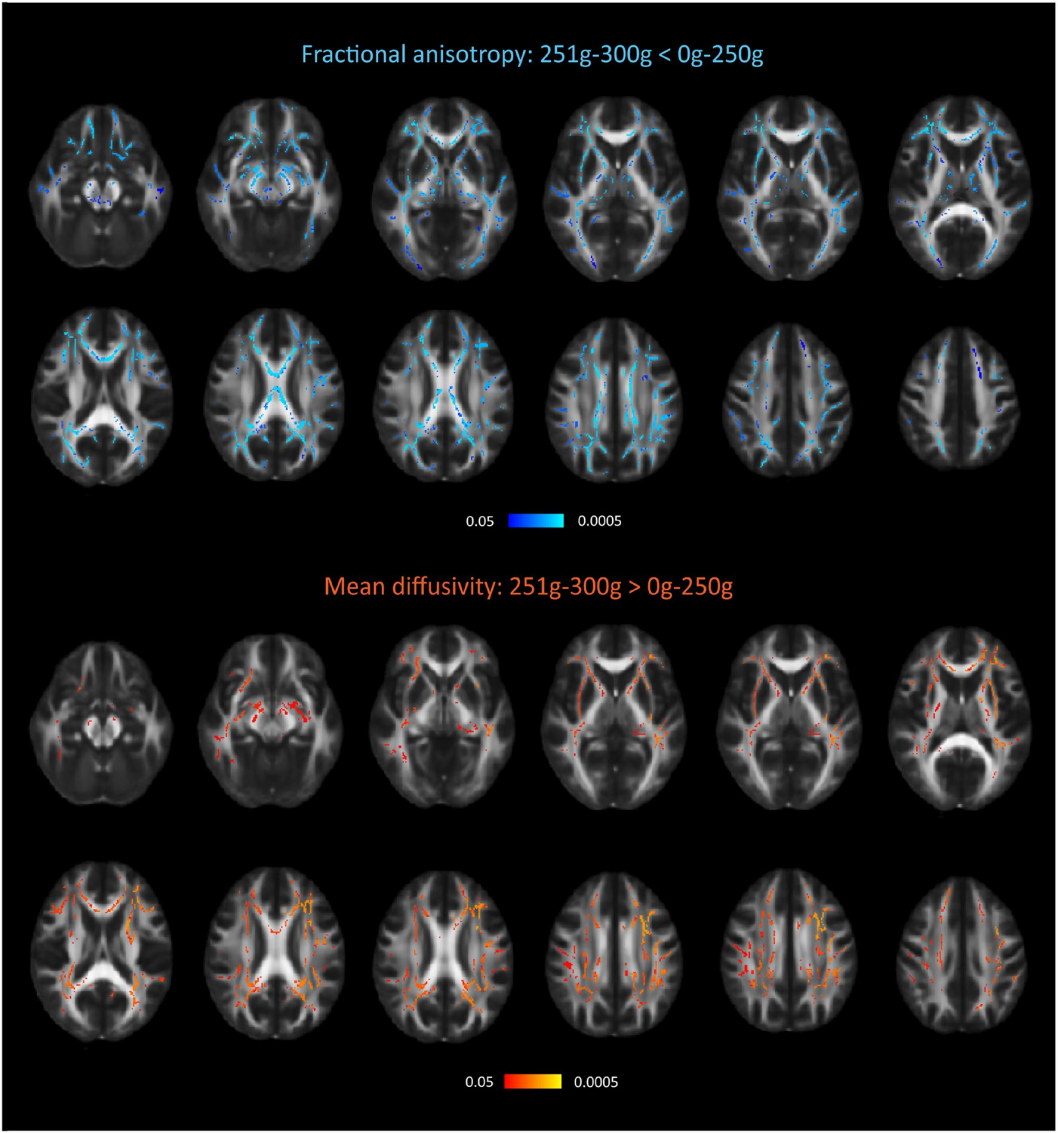
The reduction of fractional anisotropy and increase of mean diffusivity in participants who consumed > 250 g/week compared with participants who consumed ≤ 250 g/week. Reduced FA and increased MD in participants who consumed > 250 g/week compared with participants who consumed ≤ 250 g/week. Cold colors, areas of significant reduction, darkest blue = *p* < 0.05 brightest blue = *p* < 0.005. Warm colors, areas of significant increase, darkest red = *p* < 0.05 brightest red = *p* < 0.005

Comparing participants consuming ≤ 250 g/week with participants consuming > 250 g/week (*n* = 41) we found reduced FA and increased MD in a large number of tracts including anterior thalamic radiation, corticospinal tract, cingulum, forceps major, forceps minor, inferior fronto-occipital fasciculus, inferior longitudinal fasciculus, superior longitudinal fasciculus, uncinate fasciculus, and superior longitudinal fasciculus (Fig. 4, Supplementary table 6).

## Discussion

In the present study, we aimed to examine the association between current alcohol consumption and brain gray and white matter structure in a large population-based sample of 70-year-olds.

Alcohol consumption of 250 g pure alcohol or more per week was predominantly associated with atrophy of the PFC. These results are in line with a longitudinal study that found increased alcohol-related cortical atrophy rate in frontal > parietal > insular > cingulate and temporal cortex [36]. However, in contrast to Sullivan et al. [35], we also observed thinning in the right occipital cortex. Further, Sullivan et al. (2019) reported that volume loss in the precentral gyrus and the superior frontal gyrus was higher in patients with AUD at 30 years of age, and that the maximal difference in cortical thickness in these regions was found when 70-year-old patients with AUD were compared with 70-year-old controls (ibid). Thus, our results confirm this previous study and indicate that the precentral and superior frontal gyrus may be particularly (and early) affected by alcohol.

Consistent with previous results, we found predominantly right-sided in high-consuming participants. Many studies have found more alcohol-related cortical atrophy in the right than left hemisphere, which led some authors to propose a “right hemisphere hypothesis” [28]. This hypothesis is further supported by the observation that cognitive functions localized within the right hemisphere are more affected in patients with AUD than functions localized to the left hemisphere [10, 41].

In the present study, 25 men did consume more than 300 g per week. In addition to frontal atrophy these participants further displayed volume loss in regions known to become atrophic in Alzheimer’s disease such as the bilateral medial temporal lobe (the entorhinal and fusiform gyrus), the posterior cingulate, precuneus, in the left superior parietal cortex and bilateral supramarginal gyrus [12]. Thus, our findings indicate that high alcohol consumption (251–300 g/week) is associated with specific frontal and occipital, < right-side pattern of cortical thinning, while very high consumption (> 300 g/week) involves thinning in the frontal, temporal, occipital, and parietal lobes.

In this sample, approximately 18% of participants had mild cognitive problems (CDR = 0.5). Comparing the 251–300 g/week consuming group with the reference group in participants with CDR = 0 revealed cortical thinning in approximately the same regions as in the whole sample (Fig. 1C and Supplementary Table 3).

These findings confirm that we can identify alcohol-related gray matter changes also in participants with no cognitive problems. The lack of associations regarding white matter changes in the higher consuming groups among participants with CDR = 0 may be due to low statistical power.

While high alcohol consumption was linked to a regional specific pattern of cortical gray matter atrophy, the white matter changes were less specific. Instead, it was widely distributed over in many tracts of the brain. Potentially may this be the “signature” of alcohol-related white matter changes in non-demented individuals.

We found no white matter changes in the highest-consuming group (> 300 g/week), while the second highest (251–300 g/week) had changes in many tracts of the brain. It is difficult to explain this finding. Possibly, the splitting of the high consuming group > 250 g into 251–300 g/week vs. > 300 g/week may have reduced our statistical power so that we were not able to detect subtle changes. Such interpretation is supported by the fact that we found significant changes in white matter when the > 250 g/week group was compared with the ≤ 250 g/week group.

One strength of this study is that results are based on data from a systematically selected sample of 70-year-olds from a general population. Obviously, as all participants are 70 years of age, we do not have the confounding effect of age in our results. This is also a limitation as we cannot study whether our results would be generalizable to other age groups. On the other hand, as discussed above, the pattern of alcohol-related cortical thinning is in agreement with some other studies including middle aged or even younger subjects.

One limitation is that alcohol consumption categories are based on past month consumption.

Certainly, in some individuals, the past months may not reflect the average consumption over an extended period of time. Furthermore, current data do not allow us to analyze drinking behavior, e.g., we cannot identify individuals with alcohol addiction among the high consumers of our sample. However, considering the observed thinning in cortical areas of the frontal lobe, we can at least say that these high consumers have a non-healthy consumption pattern.

Another limitation is that while our sample is large, we still suffer from the lack of statistical power when dividing the sample into seven groups. The reason for doing this was that we aimed to find the cutoff value for how much alcohol that can be consumed before structural brain changes in terms of reduced cortical thickness or reduced FA in whiter matter occur. The negative effect of alcohol consumption was found to occur in a non-linear fashion, in which consumption above 250 g per week was linked to significant brain changes.

A final limitation is that we have not assessed comorbid conditions, such as psychiatric disorders, that potential may be associated with brain atrophy. While one previous study indicates that such factors could be interesting to consider [40], we aim to address this in a follow-up study.

## Conclusions

In conclusion, we found alcohol-related cortical thinning and white matter changes in 70-year-old men and women consuming more than 250 g pure alcohol per week. Cortical changes were mainly observed in the frontal lobe predominantly in the right hemisphere, while high consumption seems to affect both anterior and posterior white matter tracts in the whole brain. Our findings indicate that there is an increased risk for brain damage also among non-dependent individuals with high weekly consumption.

### Supplementary Information

Below is the link to the electronic supplementary material.

Supplementary file1 (DOCX 590 KB)

## Data Availability

Data described in the manuscript, code book, and analytic code will be made available upon request pending application and approval by the steering group of the studies, and that it is in accordance with rules and regulations from the European Union (e.g. GDPR) and Swedish law.
