# Supplementary material for: Effects of current alcohol use on brain volume among older adults in the Gothenburg H70 Birth Cohort study 2014–16
Source: Eur Arch Psychiatry Clin Neurosci. 2023 Sep 19;274(2):363–73. doi: 10.1007/s00406-023-01691-x (PMC10914911; doi:10.1007/s00406-023-01691-x)
Supplement: Supplementary file 1 — Supplementary file1 (DOCX 590 KB) [file 406_2023_1691_MOESM1_ESM.docx]

**Supplementary data**

**Flow-chart for the included sample**


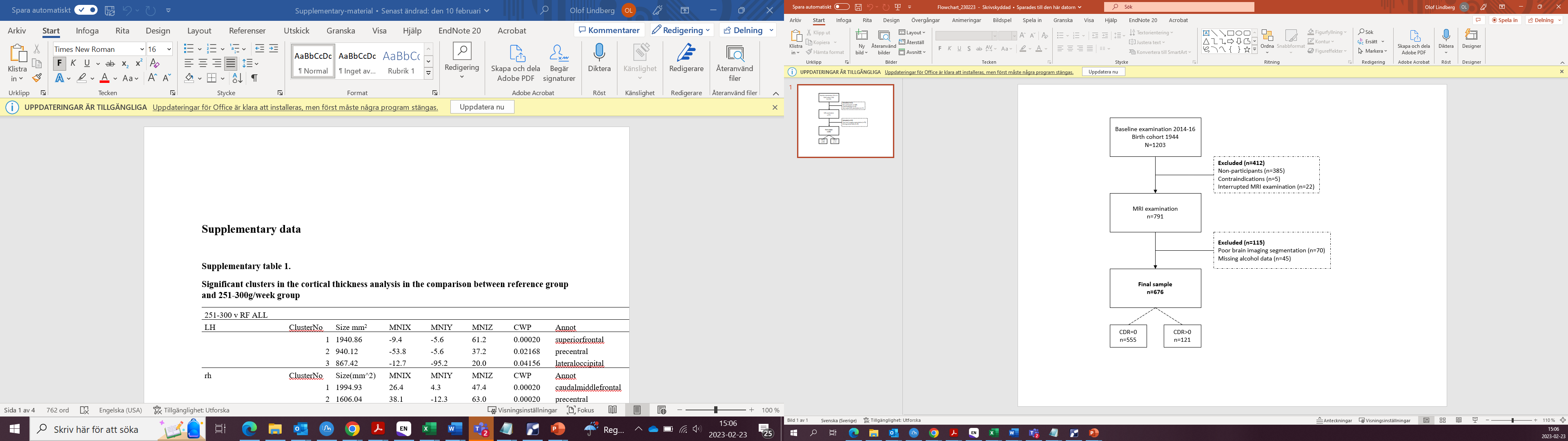


**Supplementary table 1.**

**Significant clusters in the cortical thickness analysis in the comparison between reference group and 251-300g/week group**

| 251-300 v RF ALL |  |  |  |  |  |  |  |  |
| --- | --- | --- | --- | --- | --- | --- | --- | --- |
| LH | ClusterNo | Size mm^2^ | MNIX | MNIY | MNIZ | CWP | Annot |  |
|  | 1 | 1940.86 | -9.4 | -5.6 | 61.2 | 0.00020 | superiorfrontal | |
|  | 2 | 940.12 | -53.8 | -5.6 | 37.2 | 0.02168 | precentral | |
|  | 3 | 867.42 | -12.7 | -95.2 | 20.0 | 0.04156 | lateraloccipital | |
| rh | ClusterNo | Size(mm^2) | MNIX | MNIY | MNIZ | CWP | Annot |  |
|  | 1 | 1994.93 | 26.4 | 4.3 | 47.4 | 0.00020 | caudalmiddlefrontal | |
|  | 2 | 1606.04 | 38.1 | -12.3 | 63.0 | 0.00020 | precentral | |

**Supplementary table 2.**

**Significant clusters in the comparison between participants that consumed 0-250g/wk vs. participants that drank >250g/wk**

|  | ClusterNo | Size mm^2^ | MNIX | MNIY | MNIZ | CWP | Annot |
| --- | --- | --- | --- | --- | --- | --- | --- |
| lh | 1 | 2036.02 | -13.0 | 32.3 | 43.7 | 0.00010 | superiorfrontal |
| rh | 1 | 1298.03 | 25.4 | 3.9 | 43.0 | 0.00210 | caudalmiddlefrontal |
|  | 2 | 885.88 | 29.7 | -77.1 | 16.6 | 0.03380 | lateraloccipital |
|  | 3 | 850.56 | 36.9 | -9.3 | 58.7 | 0.04290 | precentral |

lh, left hemisphere; rh, right hemisphere; Size, size in mm^2^; MNIX, MNIY, MNIZ; MNI coordinates; CWP, clustervise p-value; Annot, annotation of region.

**Supplementary table 3.**

**Significant clusters in the cortical thickness analysis in the comparison between men in the reference group and men in the 251-300g/week group with CDR=0**

| 251-300 v RF CDR=0 | | | | | | | | |
| --- | --- | --- | --- | --- | --- | --- | --- | --- |
| LH | ClusterNo | Size mm2 | MNIX | MNIY | MNIZ | CWP | Annot |  |
|  | 1 | 879.10 | -53.2 | -5.7 | 36.5 | 0.03842 | precentral | |
| RH | ClusterNo | Size(mm^2) | MNIX | MNIY | MNIZ | CWP | Annot |  |
|  | 1 | 2050.53 | 26.0 | 3.8 | 47.3 | 0.00020 | caudalmiddlefrontal | |
|  | 2 | 1459.60 | 47.2 | -7.5 | 35.0 | 0.00040 | precentral | |

**Supplementary table 4.**

**Significant clusters in the cortical thickness analysis in the comparison between men in the reference group and men in the >300g/week group**

| Above 300g v RF men | | | | | | | | |
| --- | --- | --- | --- | --- | --- | --- | --- | --- |
| lh | ClusterNo | Size mm^2^ | MNIX | MNIY | MNIZ | CWP | Annot |  |
|  | 1 | 3520.14 | -46.2 | -29.5 | 55.5 | 0.00020 | postcentral | |
|  | 2 | 3233.20 | -33.8 | -48.8 | -8.3 | 0.00020 | fusiform |  |
|  | 3 | 3210.96 | -4.3 | -27.5 | 33.4 | 0.00020 | posteriorcingulate | |
|  | 4 | 1089.60 | -20.7 | 7.7 | 48.8 | 0.00858 | superiorfrontal | |
|  | 5 | 886.99 | -36.2 | -30.9 | 21.9 | 0.03489 | supramarginal | |
| rh | 1 | 5024.19 | 57.5 | -22.0 | 28.2 | 0.00020 | supramarginal | |
|  | 2 | 2636.40 | 29.1 | -57.8 | -14.8 | 0.00020 | fusiform |  |
|  | 3 | 1523.11 | 43.9 | -22.6 | 3.4 | 0.00020 | transversetemporal | |
|  | 4 | 1451.15 | 12.9 | -19.0 | 38.0 | 0.00060 | posteriorcingulate | |

**Supplementary table 5.**

**Significant differences between groups with distinct alcohol consumption habits in fractional anisotropy and mean diffusivity maps in the whole sample**

| **Higher fractional anisotropy in 0-50g compared to the 151-200g group** | | | | |
| --- | --- | --- | --- | --- |
| Cluster size | Max X | Max Y | Max Z | Tracts * |
| 422 | -17 | 15.3 | 33.4 | Body of corpus callosum, Superior corona radiata |
| **Higher fractional anisotropy in 0-50g compared to the 251-300g group** | | | | |
| Cluster size | Max X | Max Y | Max Z | Tracts * |
| 27190 | 7 | -6.69 | 20.3 | Corpus callosum, Anterior and posterior limb of internal capsule, Retrolenticular part of internal capsule, Anterior and superior corona radiata, Posterior thalamic radiation, Sagittal stratum, External capsule, Cingulum, Superior longitudinal fasciculus |
| **Lower mean diffusivity in 0-50g compared to the 251-300g group** | | | | |
| Cluster size | Max X | Max Y | Max Z | Tracts * |
| 11541 | 1 | -28.8 | 21 | Corpus callosum, Anterior and posterior limb of internal capsule, Retrolenticular part of internal capsule, Anterior and superior corona radiate, Posterior thalamic radiation, External capsule, Superior longitudinal fasciculus |
| 6674 | -41 | 26 | 20.6 | Corpus callosum, capsule, Retrolenticular part of internal capsule, Anterior and superior corona radiate, Posterior thalamic radiation, External capsule |

* Only tracts with > 1% probability are included.

**Supplementary Table 6.**

**Significant differences between groups with distinct alcohol consumption habits in fractional anisotropy and mean diffusivity maps in the whole sample**

| **Lower fractional anisotropy in 251-300g compared to the 0g-250g group** | | | | |
| --- | --- | --- | --- | --- |
| Cluster size | Max X | Max Y | Max Z | Tracts * |
| 43728 | -0.355 | -12 | 13.1 | Anterior thalamic radiation, Corticospinal tract, Cingulum (cingulate gyrus), Forceps major, Forceps minor, Inferior fronto-occipital fasciculus, Inferior longitudinal fasciculus, Superior longitudinal fasciculus, Superior longitudinal fasciculus, Uncinate fasciculus, Superior longitudinal fasciculus (temporal part) |
| 373 | 22.3 | -83.3 | 4.56 | Forceps major,  Inferior fronto-occipital fasciculus,  Inferior longitudinal fasciculus |
| 163 | -52.9 | -21.9 | -15.7 | Inferior longitudinal fasciculus,  Superior longitudinal fasciculus |
| 137 | -13.7 | 28.8 | 42.1 | Cingulum (cingulate gyrus),  Inferior fronto-occipital fasciculus, Superior longitudinal fasciculus, Superior longitudinal fasciculus (temporal part) |
| 13 | -57.1 | -37.2 | -5.15 | Superior longitudinal fasciculus,  Superior longitudinal fasciculus (temporal part) |
| 12 | -55 | -32.9 | -8.67 | Superior longitudinal fasciculus,  Superior longitudinal fasciculus (temporal part) |
| **Higher mean diffusivity in 251-300g compared to the 0g-250g group** | | | | |
| Cluster size | Max X | Max Y | Max Z | Tracts * |
| 26598 | -0.808 | -10.5 | 19.8 | Anterior thalamic radiation,  Corticospinal tract, Cingulum (cingulate gyrus), Forceps major, Forceps minor, Inferior fronto-occipital fasciculus,  Inferior longitudinal fasciculus,  Superior longitudinal fasciculus,  Uncinate fasciculus, Superior longitudinal fasciculus (temporal part) |

* Only tracts with > 1% probability are included.

**Supplementary Figure 1.** No difference between men and women in cortical areas that were atrophic in the 251-300g/week-group


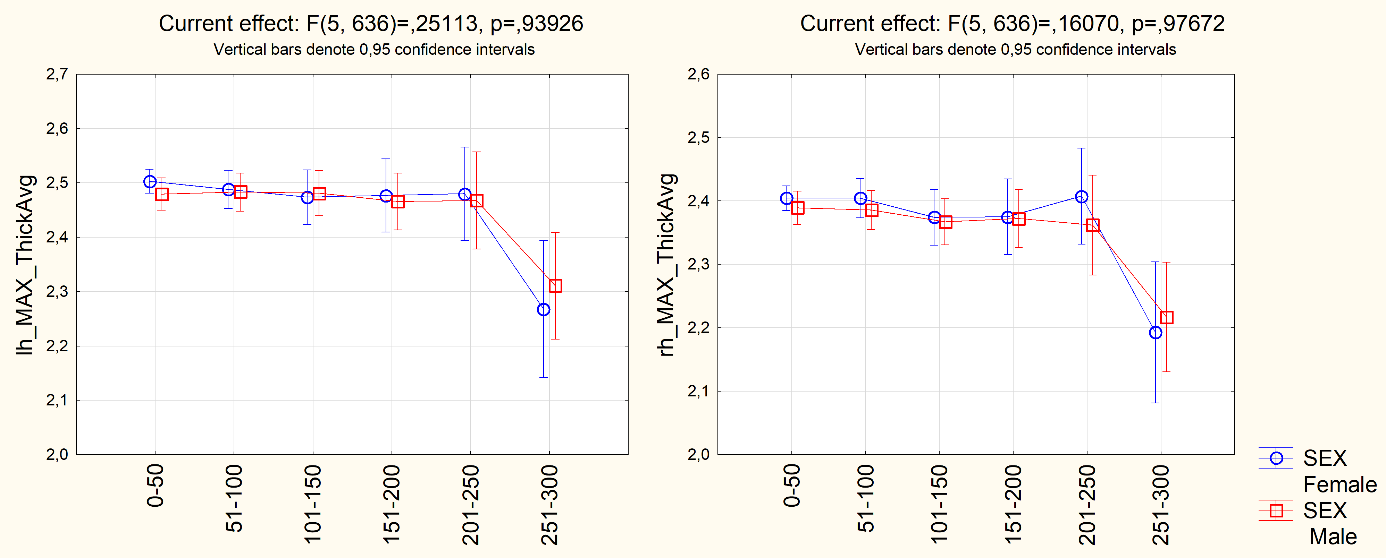
The graph denotes cortical thickness under the area that were atrophic in participants that drank more than 250g/week. Blue color, females; red color, males.
